# Odd-skipped and Stripe act downstream of Notch to promote the morphogenesis of long appendicular tendons in *Drosophila*

**DOI:** 10.1242/bio.038760

**Published:** 2019-03-22

**Authors:** Lilia Laddada, Krzysztof Jagla, Cédric Soler

**Affiliations:** GReD Laboratory, Clermont-Auvergne University, INSERM U1103, CNRS UMR6293, 63000 Clermont-Ferrand, France

**Keywords:** Tendon, *Odd-skipped*, Tubulogenesis, *Drosophila* leg, Notch, Muscle development

## Abstract

Multiple tissue interactions take place during the development of the limb musculoskeletal system. While appendicular myogenesis has been extensively studied, development of connective tissue associated with muscles has received less attention. In the developing *Drosophila* leg, tendon-like connective tissue arises from clusters of epithelial cells that invaginate into the leg cavity and then elongate to form internal tube-shape structures along which muscle precursors are distributed. Here we show that stripe-positive appendicular precursors of tendon-like connective tissue are set up among intersegmental leg joint cells expressing *odd-skipped* genes, and that Notch signaling is necessary and locally sufficient to trigger stripe expression. This study also finds that *odd-skipped* genes and *stripe* are both required downstream of Notch to promote morphogenesis of tube-shaped internal tendons of the leg.

## Abstract (precis)

**Summary:** In this paper, we show that Notch promotes the tendon development by inducing Stripe expression in leg discs and that both Stripe and Odd-skipped are required to form tube-like tendons.

## INTRODUCTION

Muscle-associated connective tissues (CTs), including tendons, are essential to muscle patterning and transmission of contraction forces to the skeleton. In addition to their structural and physiological roles, there is growing evidence that CTs are also an important source of extrinsic cues regulating skeletal muscle organization, growth, differentiation and regeneration (Hasson, 2011; Huang, 2017; Nassari et al., 2017). However, the molecular mechanisms underlying CT specification and differentiation have not been completely elucidated.

In *Drosophila*, somatic muscles are anchored to the exoskeleton via specialized tendon-like ectodermal cells called muscle attachment sites (MASs) or apodemes. Larval muscle extremities are connected to a single MAS characterized by the expression of the zinc-finger transcription factor Stripe (Volk, 1999), whose vertebrate orthologs Egr1/2 also play a role during tendon differentiation (Lejard et al., 2011). Stripe (Sr) is the earliest known marker of MAS precursors and is essential to induce the differentiation of all tendon-like cells, including adult tendon precursors (Frommer et al., 1996; Fernandes et al., 1996; Vorbrüggen and Jäckle, 1997; Ghazi et al., 2003; Soler et al., 2004). Thus to allow the establishment of a correct stereotyped muscle pattern, location of Sr-expressing cells has to be precisely defined.

In the embryo, while late expression of the SrA isoform relies on interaction with muscle fibers (Becker et al., 1997; Volohonsky et al., 2007), initial induction of the SrB isoform is triggered by a combination of ectodermic signals: Hedgehog (Hh), Wnt/Wingless and Spitz/EGF respectively (Hatini and DiNardo, 2001; Piepenburg et al., 2000). In the wing disc notum, multiple regulatory elements including Notch, Wnt and Dpp pathways cooperate to induce and maintain five distinct Sr-positive domains forming flight MASs (Fernandes et al., 1996; Ghazi et al., 2003; Usui et al., 2004). In the leg disc, seven clusters of Sr-expressing cells are specified between third instar larval and early pupa stage. They are distributed along the proximodistal axis at stereotyped dorso-ventral and antero-posterior positions. Remarkably, these Sr-positive clusters undergo invagination and elongate to form long internal tube-shaped tendons (Soler et al., 2004). It has been suggested that tendon precursors provide positional information to adjacent muscle founders, as disturbing tendon development affects spatial distribution of myoblasts (Maqbool et al., 2006; Soler et al., 2016). However, the mechanisms regulating *stripe* gene expression in the leg disc and governing the development of these unique internal tendons remain to be elucidated.

Here we show that tendon precursors are selected among narrow bands of cells expressing *odd-skipped* (*odd*) gene family members. *Odd* genes encode zinc-finger transcription factors acting downstream of the Notch pathway to control local invagination/folding of the leg disc to form the future joints between leg segments (Hao et al., 2003; de Celis Ibeas and Bray, 2003). In the absence of Sr, Odd expression is not affected, but presumptive tendon cells, after initiating invagination, do not form tube-like structures, indicating that both Sr and Odd are required for development of long internal tendons of the leg. Because Notch initiates Sr expression in a subset of Odd-positive joint cells, we infer that it plays a pivotal role in appendicular CT specification and morphogenesis by making joint cells competent to develop into tube-like internal tendons.

## RESULTS AND DISCUSSION

### Stripe is expressed in a subset of Notch activated cells from true joints

**Fig. 1.**
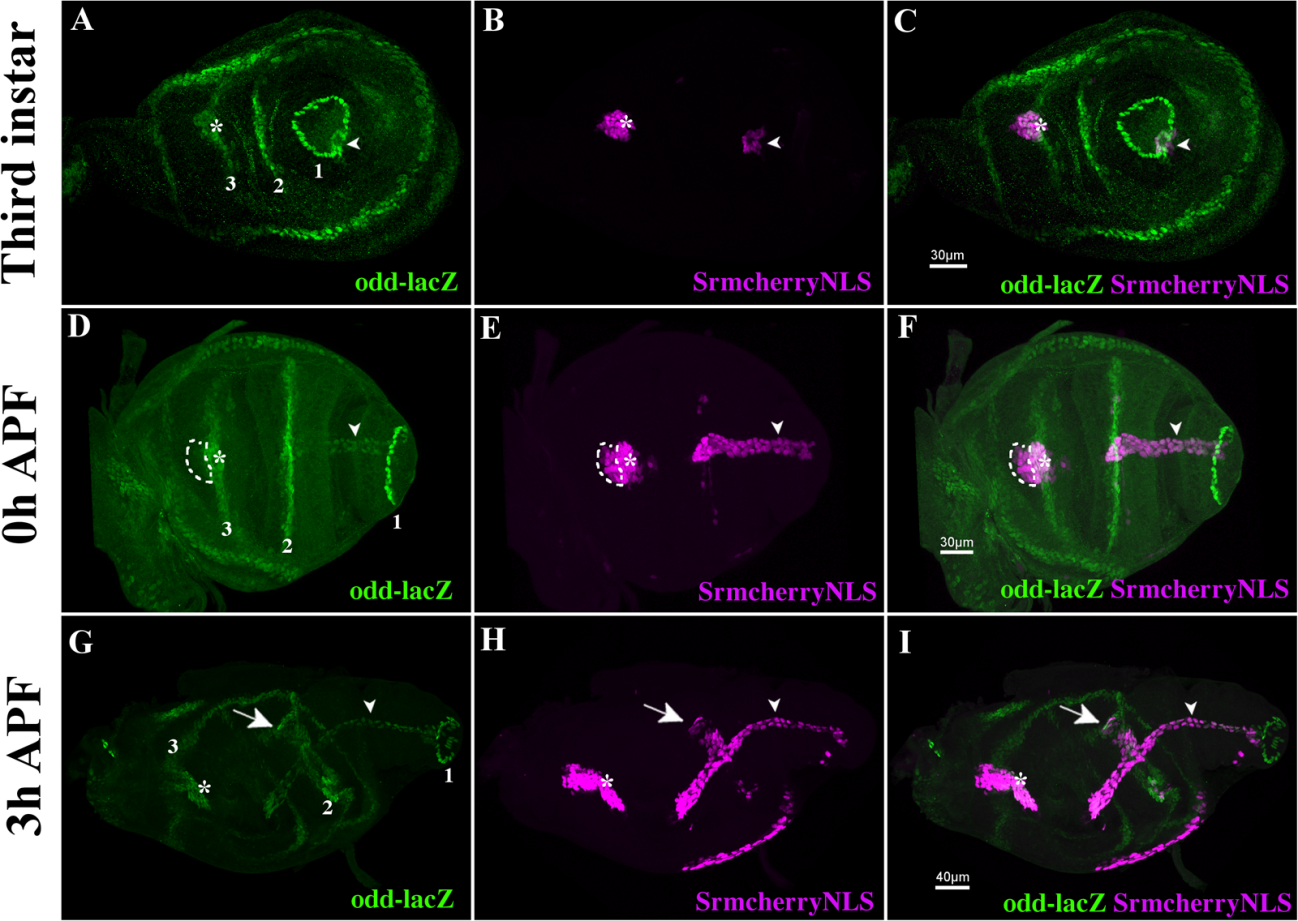
**Tendon precursors arise from Odd-skipped positive cells.** Confocal images of leg disc tendons are revealed by Stripe-Gal4>UAS-mCherryNLS (magenta) and segmental true joints by Odd^RK11lacZ^ expression (green). (A–C) Selected optical sections of L3 disc, Sr>mCherry expression is detected among rings of Odd-expressing cells of pre-tarsus/T5 joint (ring 1, arrowheads) and femur/tibia dorsal junction (ring 3, asterisks). Remarkably, at pre-tarsus/T5 junction, Sr>GFP-positive cells form a ring surrounding a lumen (arrowheads) prefiguring the formation of the long tendon of the tarsi. Note that only cells at the surface are visible on these sections and no Sr>mCherry cells are detected along the T1/tibia true joint at this stage (ring 2). (D–F) Leg discs at the beginning of pupation, long tendons of the tarsi have grown deeply into the leg cavity (arrowheads). The number of Sr>mCherry cells in the dorsal femur have increased (asterisks), some of these cells expressed no or very low levels of Odd-lacZ (dashed outlined areas). Note the presence of a few Sr>mCherry cells along the T1/tibia joint (ring 2). (G–I) On everting leg disc at 3 h after pupae formation (APF), all tendon precursors were specified and co-expressed Sr>mCherry and Odd-lacZ. Sr>mCherry cells (arrowheads), arising from pre-tarsus/T5 joint (ring 1 in G), have deeply invaginated to form the long internal tendon of the tarsi. Tendon precursor (asterisks) associated with the femur/tibia joint (ring 3 in G) is beginning to invaginate. Note the nascent tendon precursor expressing Sr>mCherry (arrows) along the tibia/T1 joint (ring 2 in G).

**Fig. 2.**
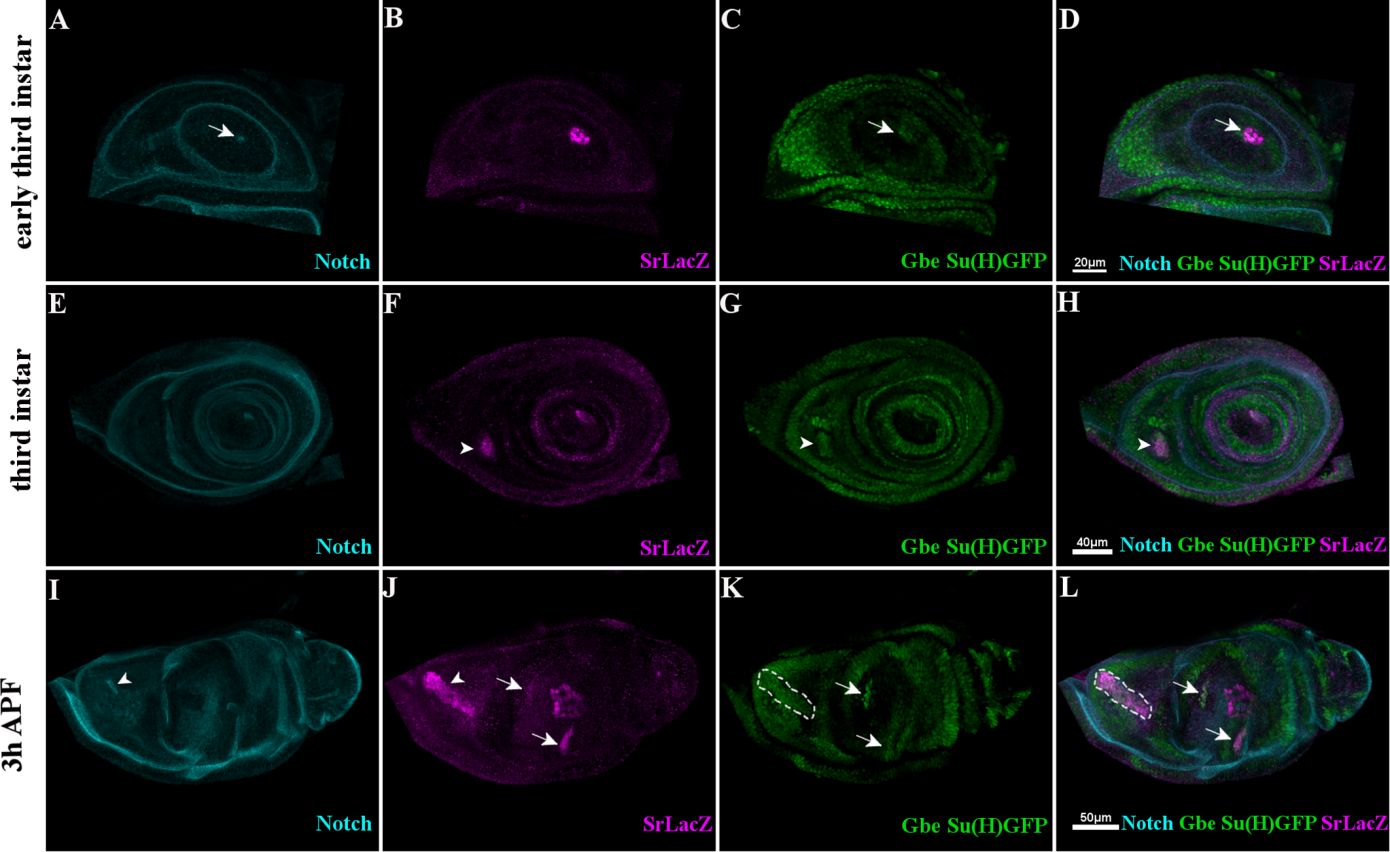
**Notch pathway activity in tendon precursors.** Expression of Notch (cyan), Sr-lacZ (magenta) and Gbe-Su(H)-GFP (green) in leg discs. (A–D) Early L3, Notch (arrow in A) is first detected at the apical surface of Sr-LacZ cells corresponding to the future tarsi long tendon (B); these cells express Gbe-Su(H)-GFP, Notch pathway activity reporter (arrows in C and D). (E–H) From mid L3, Sr-lacZ accumulates in the developing tendon of the dorsal femur following the expression of Notch protein and Gbe-Su(H)-GFP (arrowheads). (I–L) 3 h APF, in selected optical sections, Sr-LacZ cells invaginate in the dorsal femur to form long tendon accumulating Notch proteins at the apical surface (arrowheads in I and J); these cells show Notch-positive activity (dashed outlined areas in K and L). Note the appearance of later tendon precursors in other segments also accumulating Gbe-Su(H)-GFP expression (arrows in J–L).

The *Drosophila* leg is composed of nine segments separated by joints shaped by constriction/folding of cells forming a concentric ring between each leg segment. ‘True’ joints separate segments from the coxa-trochanter to the tibia-tarsi (T1) junctions and between the tarsus (T5) and pre-tarsus (claws). They are characterized by the expression of the four *odd-skipped* family genes: *odd-skipped*, *sob*, *drm* and *bowl* (Hao et al., 2003; de Celis Ibeas and Bray, 2003; Levine et al., 1997) and the insertion of internal tendons to which the leg muscle fibers are attached (Fristrom and Fristrom, 1993; Mirth and Akam, 2002). Upon Notch pathway activation at segmental boundaries by its ligands Delta/Serrate, Odd transcription factors induce invagination of joint cells (Hao et al., 2003; de Celis Ibeas and Bray, 2003; Bishop et al., 1999; Celis et al., 1998; Mishra et al., 2001; Rauskolb, 2001; Rauskolb and Irvine, 1999). We had previously shown that most Sr-positive cells co-express Odd-skipped so that internal leg tendons could arise from cells that are part of the segmental joints (Soler et al., 2004). To explore this possibility, we further characterized Sr expression at different stages of leg disc development with respect to Odd-skipped expression (Fig. 1) using the Sr-Gal4>UAS-mCherryNLS transgenic line crossed with a line carrying *odd-lacZ*^rK111^ allele (de Celis Ibeas and Bray, 2003). During the third instar, five distinct rings of Odd-lacZ expression appear at the position of presumptive true joints (Fig. 1 and Hao et al., 2003). The tendon that forms the earliest is the long tendon of the tarsi that arises from cells co-expressing Sr and Odd-lacZ at the T5/pre-tarsus boundary at early third instar (not shown and Fig. 1A–C). These cells invaginate and elongate into the tarsal cavity formed by the progressive evagination of the leg disc at the beginning of metamorphosis (Fig. 1D–F). By the end of leg development, they will form a long internal tube extending from pre-tarsus to femur with the apical pole facing the lumen (see Movie 1 and Soler et al., 2004). With a short delay, another cluster of tendon precursor cells is specified during the third instar at the femur/tibia boundary in the dorsal position (Fig. 1A–C). Subsequently, during early pupation, additional Sr-positive clusters are specified along the five segmental pre-patterned Odd-positive boundary rings (Fig. 1D–I and Soler et al., 2004). Regarding the long tendon of the tarsi, these cells undergo invagination and elongate to form an internal tube-shape tendon. These observations indicate that Odd/Sr-positive tendon precursors are specified progressively following the leg proximo-distal segmentation with Odd preceding Sr expression. Strikingly, Sr positive cells appear to express different levels of Odd-lacZ; this is particularly remarkable in the dorsal femur where the Sr positive cells, located at the aperture of the elongating tendon, show no or a faint expression of Odd-lacZ (Fig. 1D–F). As leg segmentation and expression of *odd* genes are Notch dependent (Hao et al., 2003; de Celis Ibeas and Bray, 2003**)**, we also examined Notch protein and Notch pathway activity using the *Gbe+Su(H)GFP* reporter line (de Navascués et al., 2012) combined with *sr-lacZ^03999^* line (Frommer et al., 1996; Usui et al., 2004). We observed that Notch protein localizes to the apical surface of the developing tube-shaped tendons (Figs 2A,E,I and S1, see also Movies 1 and 2). These tendon cells are also characterized by the accumulation of Gbe+Su(H)GFP (Fig. 2C,G,K). With Odd-lacZ, Notch pathway activation appears sequentially and precedes Sr expression. Of note, while tendons grow and develop, Gbe+Su(H)GFP expression progressively decreases, suggesting that Notch activation could be restricted to the early stage of tendon specification.

### Stripe acts downstream of Notch in appendicular tendon-like cells

To investigate the relevance of Notch signaling for Sr expression in tendon precursors, we first analyzed Sr-lacZ expression in a Notch-thermosensitive background (see Materials and Methods). No lacZ staining could be detected in the leg discs of N^ts^ mutants raised at non-permissive temperature (31°C) compared to control leg disc (Fig. S2). Interestingly, switching N^ts^ larvae to 31°C after the cells have been specified as tendon precursors (after Sr induction), does not affect Sr-lacZ expression. Moreover, when we attenuated Notch using Sr-Gal4 crossed with different UAS-NotchRNAi lines, none of these lines had any effect on Sr expression (not shown). This observation means that once tendon precursors have been specified (as Sr-Gal4 is active), downregulation of Notch expression has no effect on the later tendon development. We thus speculate that Notch activity is necessary to initiate Sr expression but may not be required for its maintenance and the subsequent tendon development.

**Fig. 3.**
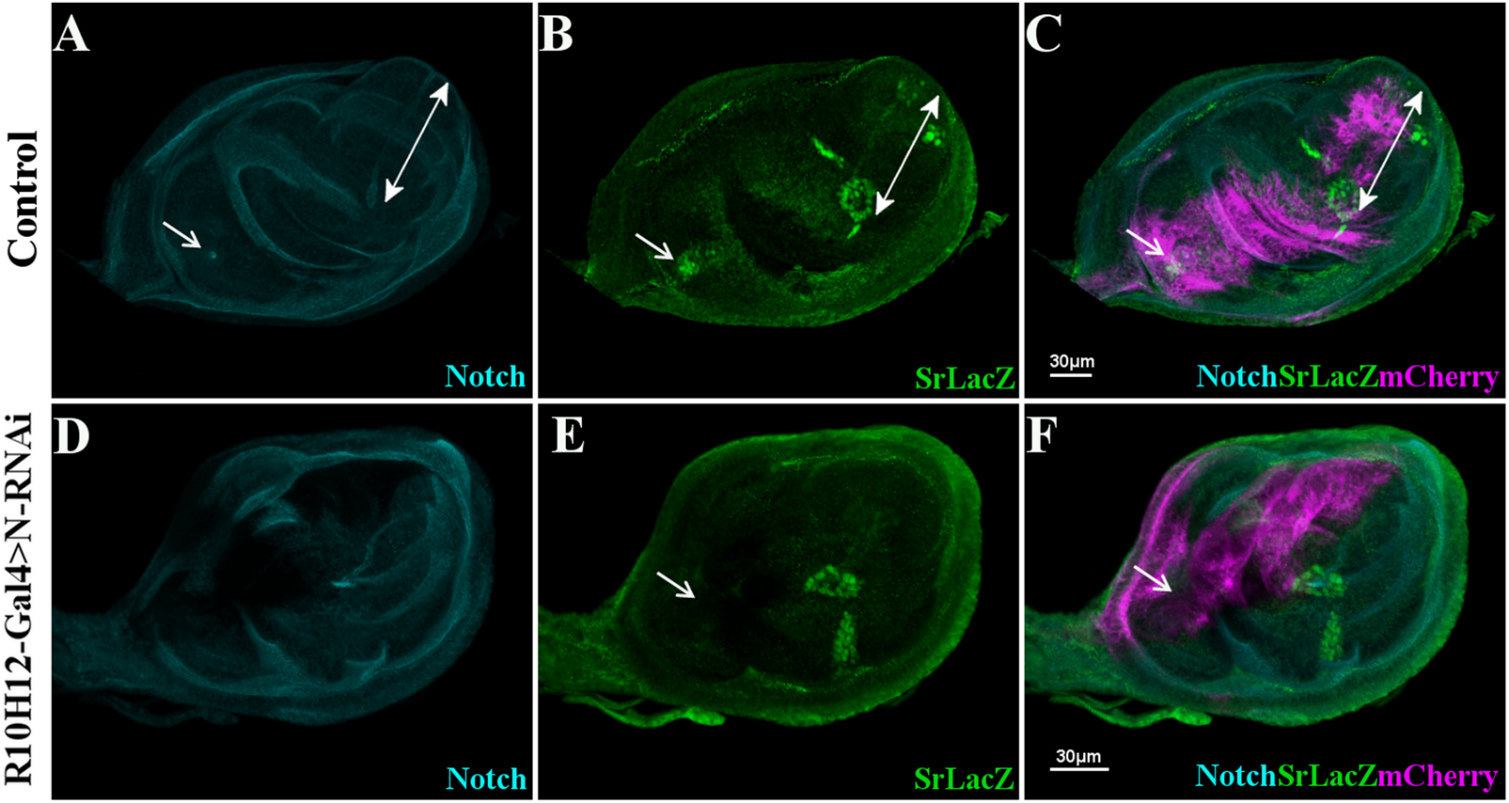
**Notch signaling is required for Stripe expression.** (A–F) Notch (cyan), Sr-lacZ expression (green) and UAS-mCherryCAAX (magenta) in R10H12-Gal4 (A–C) and R10H12-Gal4 >UAS-Notch-RNAi (D–F) leg discs at 0 h APF. (A–C) Notch is detected in invaginating tendons: selected optical sections show the tube-like shape formed by the long tendon in tarsi (double-headed arrows) and lumen aperture of the dorsal femur tendon (arrows). R10H12-gal4 pattern overlaps with the tendon precursor in dorsal femur, but not with the long tendon in tarsi (merge in C). (D–F) UAS Notch-RNAi expression strongly repressed Notch expression in R10H12-gal4 pattern (D and merge in F), including dorsal femur where Sr-lacZ expression is lost (arrows in E and F).

As the use of N^ts^ allele could primarily affect the general leg growth and segmentation, we attempted to reduce Notch function or expression within a more spatially constrained area. We combined the Sr-lacZ line with the *R10H12-Gal4* line (Jory et al., 2012; Pfeiffer et al., 2008) that drives Gal4 expression in a leg disc region overlapping the dorsal femur precursor slightly before Sr expression initiates (Fig. 3). In 0 h after pupae formation (APF) control leg discs, clusters of Sr-lacZ cells are present in the different leg segments and display accumulation of Notch protein at the apical membrane of invaginating tendon precursors (Fig. 3A). This is particularly visible for the long tendon that invaginates from the T5-pretarsus joint and crosses the tarsus segments. Cells contributing to this tendon do not express R10H12-Gal4 contrary to the Sr-lacZ positive cells of the dorsal femur as revealed by UAS-mCherryCAAX expression (Fig. 3B,C). Consequently, in *R10H12-Gal4,SrlacZ>UAS-mCherryCAAX,UAS-NotchRNAi* leg disc, Notch protein level is strongly reduced in tendon precursors of the dorsal femur and the ring of Notch accumulation formed by the invaginating tendon is no longer visible (Fig. 3D). Consequently, Sr-lacZ expression is completely abolished in these cells, whereas it remains intact in other tendons (Fig. 3E,F). To prove that endogenous Sr expression is also dependent on Notch and to exclude any RNAi off-target effects, we expressed a Dominant Negative form of Notch (*UAS-NotchDN*) using *R10H12-Gal4* (Fig. S3). We found that Sr protein was absent from the dorsal femur region and as revealed by anti-FasIII immunostaining and no invaginating structure could be detected in this part of the disc.

Overall, these results demonstrate that Notch is directly or indirectly required to initiate Sr expression in appendicular tendon precursors but is not needed for its maintenance. Through the activation of *odd* family genes (Hao et al., 2003; de Celis Ibeas and Bray, 2003), Notch also appears to be involved in making leg tendon precursors competent for invagination. However, because long internal tendons are not properly formed when invaginating cells express a dominant negative form of Sr (Soler et al., 2016), we hypothesize that the complete elongation of these tendons is Sr-dependent.

### Notch activation is locally sufficient to promote Sr expression and ectopic tendon formation

**Fig. 4.**
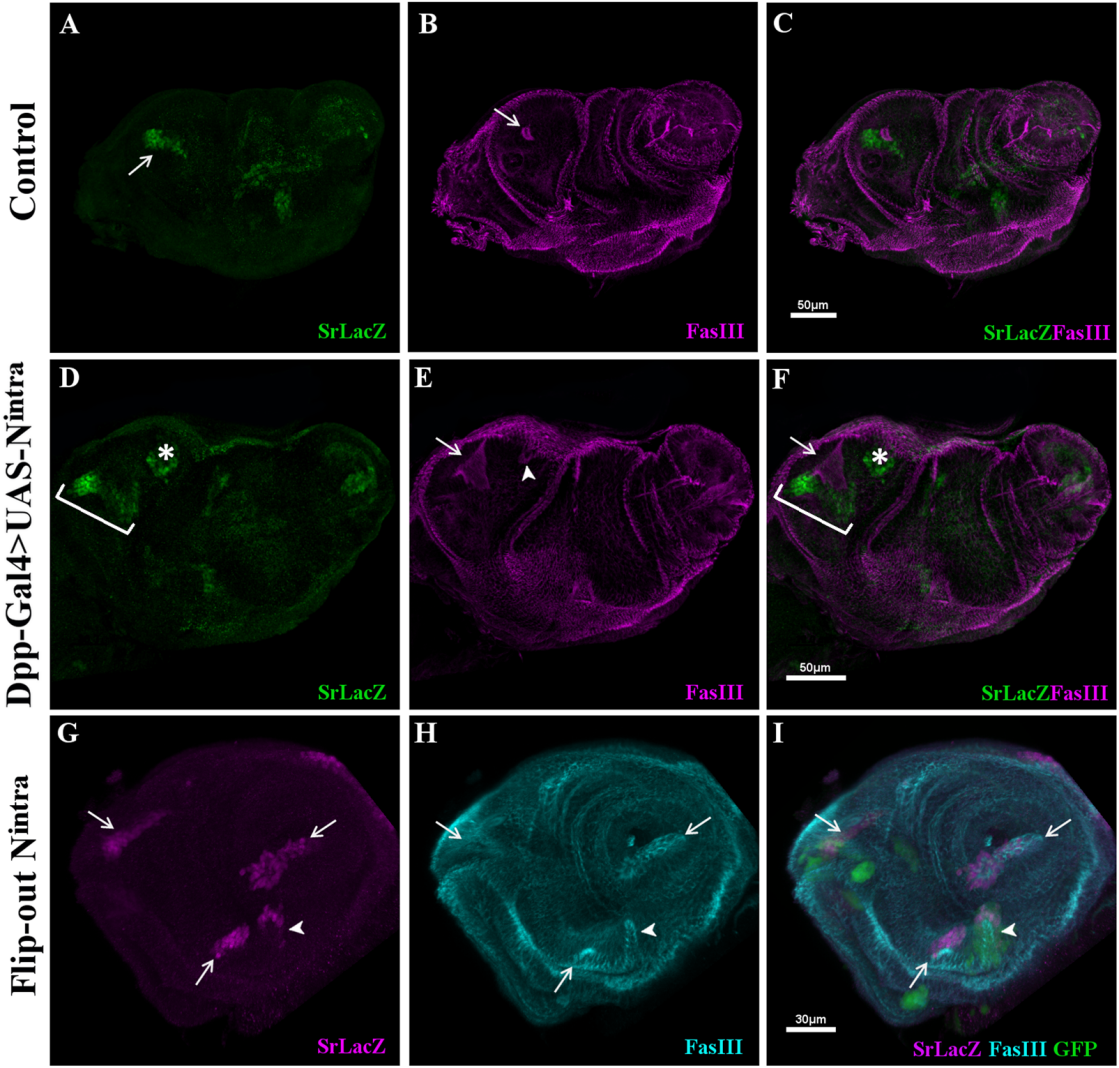
**Forced Notch pathway activation causes** **Sr-LacZ ectopic expression and ectopic tendon formation.** (A–C) Control leg disc at 0 h APF, Sr-lacZ positive cells in dorsal femur (arrow in A), surround lumen developing tendon revealed by FasIII apical accumulation (arrow in B), merged channels shown in C. (D–F) In Dpp-gal4>UAS-N^intra^ leg disc, Notch pathway activation leads to ectopic SrlacZ expression in a cluster of cells in dorsal femur (asterisks in D and F) able to invaginate (arrowhead in E). Note that more Sr-lacZ cells are recruited to form endogenous dorsal femur tendon (brackets in D and F) compared to control (A), and so these cells form a wider lumen aperture as revealed by FasIII staining (arrows in E and F). (G–I) Clone of cells expressing N^intra^ in L3 leg disc, marked by GFP expression (green in I). Sr-lacZ marked original tendon precursors (arrows) and is also induced in one of the clones in dorsal femur invaginating and starting to form an internal tube (arrowheads).

Notch signaling is known to promote joint development of the leg disc, and ectopic activation of the Notch pathway can lead to ectopic joint formation (Bishop et al., 1999; Celis et al., 1998; Rauskolb and Irvine, 1999). As we showed that tendon precursors are specified from true joint cells and that Notch is required to initiate Sr expression, we tested whether Notch could also ectopically induce Sr by expressing N^intra^ with the Dpp-Gal4 driver; which drives Gal4 expression along the A/P compartment (Staehling-Hampton et al., 1994; Morimura et al., 1996) (Fig. 4). Strikingly, we found that although Dpp-Gal4 was expressed all along the antero-dorsal domain of the leg disc, N^intra^ could mainly induce Sr-lacZ in the immediate neighborhood of endogenous Sr-lacZ-positive cells, contributing to the enlargement of the original tendon precursor. Occasionally, a new cluster of Sr-laZ-positive cells could also be detected, always in the vicinity of the dorsal tendon precursor of the femur (Fig. 4D–F). Staining of the apical surface of these cells by anti-FasIII shows that this new cluster of Sr-laZ positive cells could invaginate as an endogenous tendon (compare Figs 4A and 3D). These results suggest that only committed and/or precisely localized cells are likely to express Sr after Notch pathway activation. To confirm this possibility, we generated several small clusters of N^intra^-expressing cells using the Flip-out Gal4 technique (Ito et al., 1997). Although indentations of epithelium are often observed all along the proximo-distal axis when N^intra^ is ectopically expressed or in *odd* genes flip-out clones (Hao et al., 2003; Rauskolb and Irvine, 1999), only a few N^intra^ clones could express Sr-lacZ and elongate to create a tube-like structure (Fig. 4G,H). As these ectopic tendon-like structures are always found close to original tendon precursors, we hypothesize that tendon precursors are specified at intersections between axial signals such as Hh, Wg or Dpp (reviewed in Estella et al., 2012) and Notch/Odd+ rings defining the true joints.

### Both Odd-family and Stripe are required for tube-like tendon formation

**Fig. 5.**
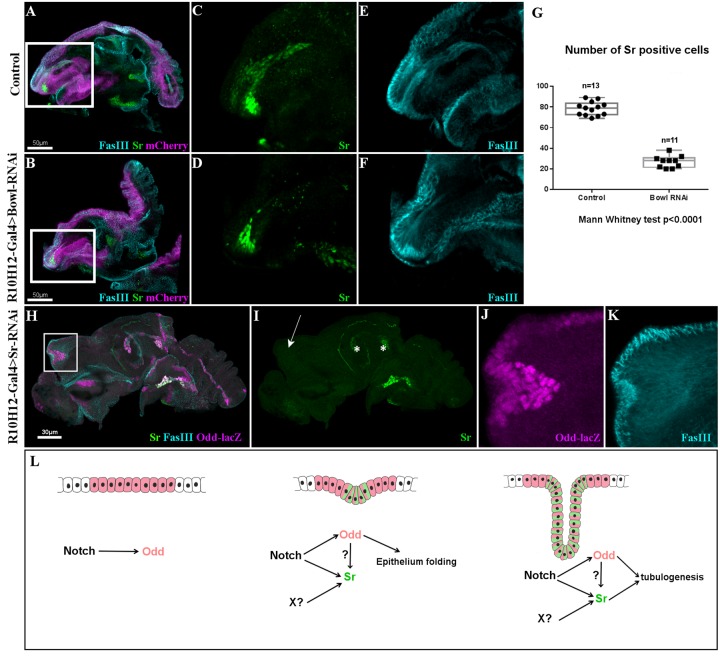
**Odd-skipped and Stripe interact to form long internal tendons.** (A–F) R10H12-Gal4>UAS-mCherryCAAX (magenta) leg discs at 5 h APF immunostained with anti-Sr (green) and anti-FasIII (cyan). (A) Control leg disc and (B) leg disc expressing UAS-BowlRNAi. (C,E) Higher magnifications from A showing Sr-expressing cells forming a long internal tube that elongates into the dorsal femur cavity. (D,F) Higher magnifications from B; number of cells expressing Sr is significantly reduced after Bowl-RNAi expression (D), remaining Sr-positive cells can still invaginate to form a tube reduced in size (compare F with E). (G) Box-plot diagram comparing number of Sr-positive cells in dorsal femur tendon in control and after expression of UAS-BowlRNAi. (H) R10H12-Gal4;Odd-lacZ>UAS-SrRNAi leg discs at 5 h APF immunostained with anti-lacZ (magenta) and anti-FasIII (cyan) and anti-Sr (green). (I) Single channel showing complete absence of Sr protein in dorsal femur where R10H12-Gal4 drives UAS-SrRNAi expression (arrow). Conversely, Sr is still expressed in other tendons (asterisks). (J,K) High magnification of dorsal femur from (H) for Odd-lacZ and FasIII channels respectively. Odd-lacZ expression is maintained in absence of Sr, FasIII accumulation at disc surface indicates that Odd-lacZ+ cells can trigger constriction of the disc epithelium but cannot form an internal tube. (M) In our model, Notch induces Odd in presumptive true joints leading to epithelium folding, Notch/Odd conjointly with unknown signal (X) trigger Sr expression responsible for tendon elongation.

As *odd* genes are targets of Notch signaling, and leg disc epithelium folding has already been described upon ectopic expressions of *odd* genes (Hao et al., 2003; de Celis Ibeas and Bray, 2003), we wondered whether the absence of these genes could affect Sr expression. Potential redundancy between different members of this family makes complete loss of function difficult to achieve, but we took advantage of an *UAS-Bowl RNAi* line predicted to have *sob* and *odd* as off-targets to reduce the expression of these three *odd* genes (Del Signore et al., 2012). In *R10H12-Gal4>UAS-Bowl-RNAi* leg discs, the number of Sr-expressing cells in the dorsal femur is severely reduced compared to controls, indicating that *odd* genes could be required for Sr expression (Fig. 5). Furthermore, when we ectopically expressed the protein lines (*R10H12-Gal4>UAS-lines*), known to interact antagonistically with Odd family proteins (Del Signore et al., 2012; Green et al., 2002; Greenberg and Hatini, 2009; Hatini et al., 2005), Sr-lacZ expression was completely abolished in the dorsal femur (Fig. S4).

One potential reason for *sr* expression being downregulated in an *odd* attenuated context is that *odd* genes could act downstream of Notch to control *sr* expression. However, in a wild-type context, not all *odd-lacZ* positive cells express *sr* (Soler et al., 2004 and this study) and more importantly, we could not observe any ectopic tendon-like structure in our most recent experiments of *odd*, *sob* or *drm* gain of functions using Dpp-Gal4 driver or Flip-out Gal4 technique (data not shown) as it is the case when we ectopically express N^intra^ (Fig. 4). Furthermore, we notice that a small group of cells still show a robust Sr expression in *bowl RNAi* context (Fig. 5). These cells appear to express a very weak level of *odd-lacZ* in control leg discs (Fig. 1) suggesting that *sr* could be expressed even in absence of *odd-skipped* genes. Thus, while we cannot rule out an indirect effect of *odd-skipped* genes' loss of function on Sr expression as this gene family is also known to repress the expression of the Notch ligand Delta (Greenberg and Hatini, 2009), we hypothesize that Notch activity is required to define a permissive zone of Odd-expressing cells giving rise to leg true joints*.* Some of these cells, under Notch and possibly Odd control, start to express Sr and form long internal tendons. To determine whether Sr is required for tube-like shaping of internal leg tendons, we prevented Sr expression by using R10H12-Gal4, which drives UAS-Sr RNAi, several hours before Sr induction in the dorsal femur (Fig. 5H–K). As shown in Fig. 5I, at 5 h APF Sr protein is completely absent in the dorsal femur while still present in other tendon precursors. In this presumptive area, where the tendon should develop, Odd-lacZ-positive cells are still present and can trigger epithelium folding, but failed to form a tube (Fig. 5J-K). Thus, Sr is required to make Odd-positive cells competent for complete invagination and formation of a tube-like structure.

In summary, previous studies and our data support a model (Fig. 4L) in which Notch signaling triggers expression of *odd* genes in rings of cells at the origin of the true joints (Hao et al., 2003; de Celis Ibeas and Bray, 2003). Along these rings, clusters of Sr-positive cells appear at different times from early L3 to the beginning of pupation in a Notch/Odd-dependent manner. This spatial and temporal restriction of Sr induction suggests that Notch signaling intersects with known local axis-defining morphogenes such as Wnt, Dpp and Hh, that are also known to regulate *sr* expression in embryonic and wing disc epithelia (Piepenburg et al., 2000; Hatini and DiNardo, 2001; Ghazi et al., 2003). Dpp and Wnt pathways act combinatorially to regulate a different set of genes in a concentration-dependent manner along the proximodistal (PD) axis (Lecuit and Cohen, 1997; Abu-Shaar and Mann, 1998). Strikingly, in our most recent experiments, downregulation of the Dpp pathway in the dorsal femur by using UAS-Smad RNAi driven by R10H12-Gal4 has only a weak effect on SrlacZ expression whereas the expression of a dominant form of dTCF totally abolished SrlacZ expression in this dorsal domain (data not shown and Fig. S5). Because Wnt and Dpp pathways are required to set up the early dorso-ventral patterning of leg discs (Lecuit and Cohen, 1997), it is difficult to draw a clear conclusion from these experiments but it appears that Wnt signaling could be one of the major pathway involved in Sr induction, concomitantly with Notch pathway.

Finally, we showed that Sr is not required to promote expression of *odd* genes and/or initial epithelium invagination, but is subsequently essential to commit Odd-positive cells as precursors of internal leg tendons able to form tube-like structures.

## MATERIALS AND METHODS

### *Drosophila* stocks and culture

The following *Drosophila* stocks were used: Dpp-gal4 (BDSC 1553), R10H12-Gal4 (Pfeiffer et al., 2008, BDSC 48278), UAS-mCherryCAAX (BDSC 59021), UAS-mCherryNLS(BDSC 38425), UAS-mCD8GFP (BDSC 32184), Gbe-Su(H)GFP (gift from S. Bray, University of Cambridge), UAS-N^intra^, UAS-Notch^DN^ (gift from S. Artavanis-Tsakonas, Harvard Medical School), UAS-NotchRNAi (BDSC 7078), UAS-BowlRNAi (VDRC 3774), UAS-lin (Greenberg and Hatini, 2009, BDSC 7074), UAS-StripeRNAi (BDSC 27701), UAS-PanDN (Van de Wetering et al., 1997, BDSC 4784) and the enhancer trap lines Sr-Gal4^md710^ (Usui et al., 2004, BDSC 2663), sr-lacZ^03999^ (BDSC 11618, Frommer et al., 1996). Odd-lacZ^rK11^ line was a gift from C. Rauskolb, Waksman Institute; this reporter transgene recapitulates expression of *odd* family genes in the developing leg disc (de Celis Ibeas and Bray, 2003). Experiments using the Gal4/UAS system (Brand and Perrimon, 1993) to induce RNAi or cDNA expression were performed at 25°C. In all experiments using RNAi to downregulate gene expression, UAS-RNAi lines were used in combination with UAS-Dicer2 allele (BDSC 24650).

For experiment using Notch^ts1^ (BDSC 2533), eggs were collected at 22°C and larvae maintained at this permissive temperature until L2. Larvae were then switched to 31°C, a restrictive temperature for Notch thermosensitive allele, until dissection.

Flip-out Gal4 clones (Ito et al., 1997) were generated using HS-Flp; Tub-STOP-Gal4, UAS-mCD8-GFP line. This line was crossed with the UAS-N^intra^ line, progeny was maintained at 25°C, and clones were induced at 48–72 h after egg laying (AEL) for 30 min at 37°C.

### Immuno-histochemistry and confocal microscopy

Staged larvae and pupae were dissected in PBS and fixed in 4% paraformaldehyde in PBS for 20 min at room temperature (for larvae and pupae up to 5 h APF). They were then stained with the following primary antibodies: rabbit anti-Twist (1/500, our lab); mouse anti-FasIII (DHSB, 1/500), chicken anti-lacZ (DHSB, 1/1000), mouse anti-N^intra^ (DHSB, 1/500) and guinea-pig anti-Stripe (1/1000, gift from T. Volk, Weizmann Institute of Science). The secondary antibodies (dilution 1/500) used were: anti-mouse Cy5, anti-chicken Cy3, anti-chicken 488, anti-rabbit Cy3 (Jackson Immunoresearch) and anti-GP 488 (Molecular Probes). Immunostaining was visualized on an inverted SP8 Leica confocal microscope, and images were analyzed with Imaris software 7.6.5.

## Supplementary Material

Supplementary information
