# Supplementary material for: Odd-skipped and Stripe act downstream of Notch to promote the morphogenesis of long appendicular tendons in Drosophila
Source: Biol Open. 2019 Mar 22;8(3):bio038760. doi: 10.1242/bio.038760 (PMC6451353; doi:10.1242/bio.038760)
Supplement: Supplementary information [file biolopen-8-038760-s1.pdf]

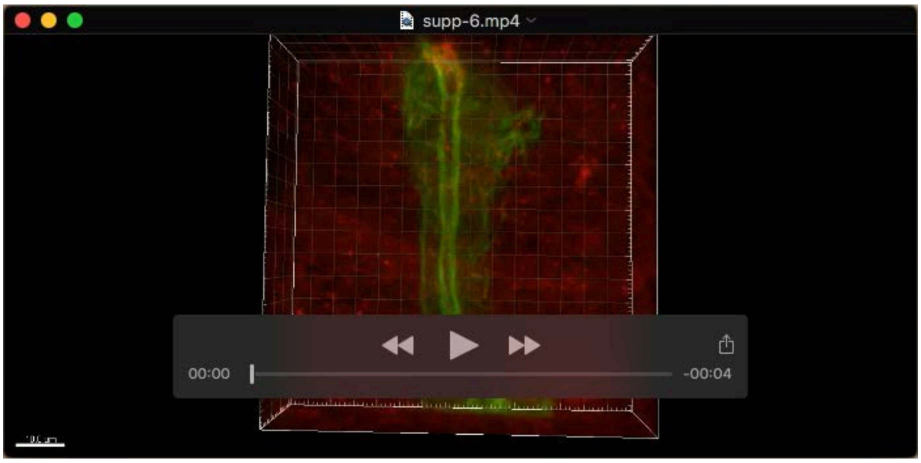


Movie 1: 3D view of the long internal tendon in tarsi of a Sr-gal4>UASLifact-GFP (green) leg disc at 0hAPF, immunostained against Nintra antibody (red).

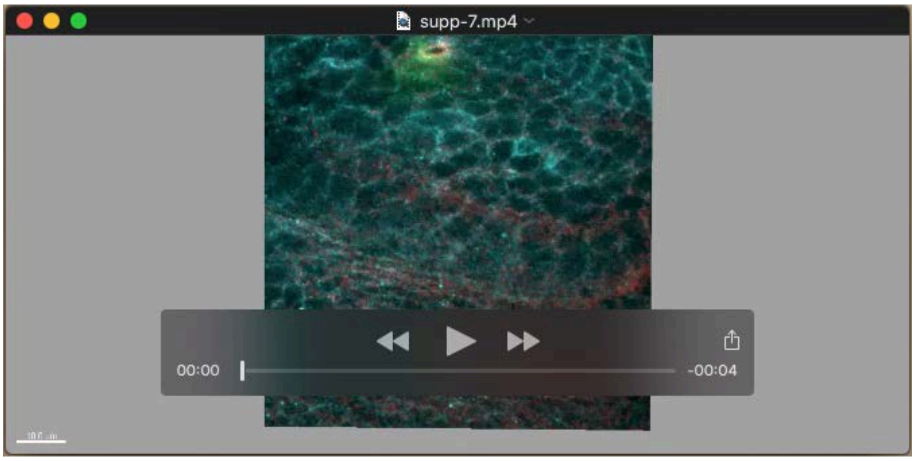


Movie 2: Z-stack confocal images showing the long tendon of the tarsi of a Sr-gal4>UASLifact-GFP (green) leg disc at 0h APF, immunostained against Nintra (red) and E-cadherin (cyan).

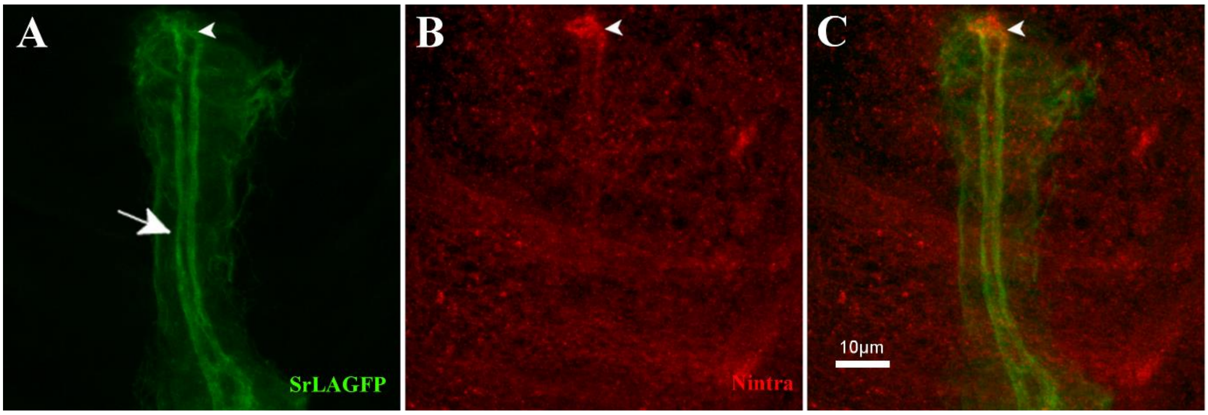


**Figure S1. Long tendons in leg disc form a tube-shape structure with Notch accumulation on apical face.**

(A-C) High magnification of a long internal tendon of the tarsi of a leg disc at 0h APF. (A) Tendon cells form a long internal tube revealed by the expression of Sr-gal4>UAS-lifactin-GFP (arrow). (B) Notch protein accumulates at the apical surface of this tube, particularly at the lumen entry (arrowhead). (C) merge.

Biology Open • Supplementary information

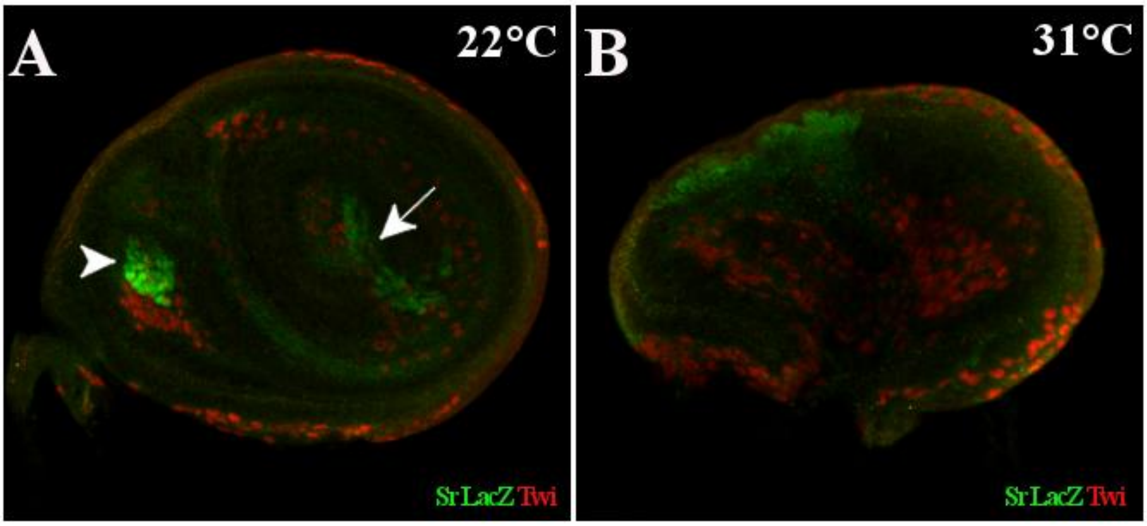


**Figure S2. Reduction of Notch pathway activity affects Stripe expression.**

(A) Sr-lacZ expression (green) in dorsal femur tendon (arrowhead) and in long tendon of tarsi (arrow) in Notch$^{ts}$ mid third instar leg disc raised at permissive temperature (22°C). (B) In leg discs raised at non-permissive temperature (31°C), Sr-lacZ expression is completely abolished, the remaining green staining corresponds to background. Staining against Twist shows myoblasts, positioning of myoblasts allows us to orientate the leg disc while A/P and D/V axis are severely affected.

Biology Open • Supplementary information

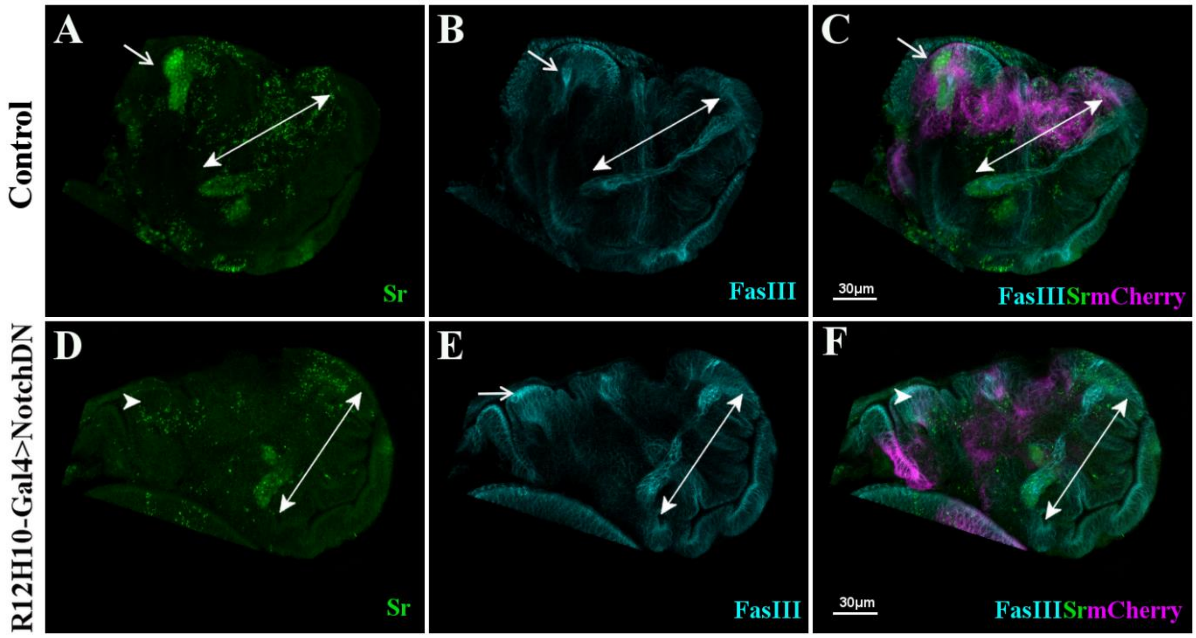


**Figure S3. Sr protein is absent when Notch signaling is affected**

Immunostaining against Stripe protein (green) and FasIII protein (cyan) in R10H12-Gal4>UAS-mCherryCAAX (magenta) leg disc at 0h APF. (A-C) In the control leg disc, tubes formed by invagination of Sr-positive cells in the dorsal femur (arrow) and tarsi (double arrow) are prominently displayed with FasIII staining. (D-F) In UAS-NotchDN disc, Sr expression is absent in the dorsal femur (arrowhead in D and F), arrow in E indicates FasIII accumulation at the apical region of cells that failed to form a tube. Sr and Fas III expressions are not affected in tarsi tendon cells that still form a long internal tube (double arrow in D-F).

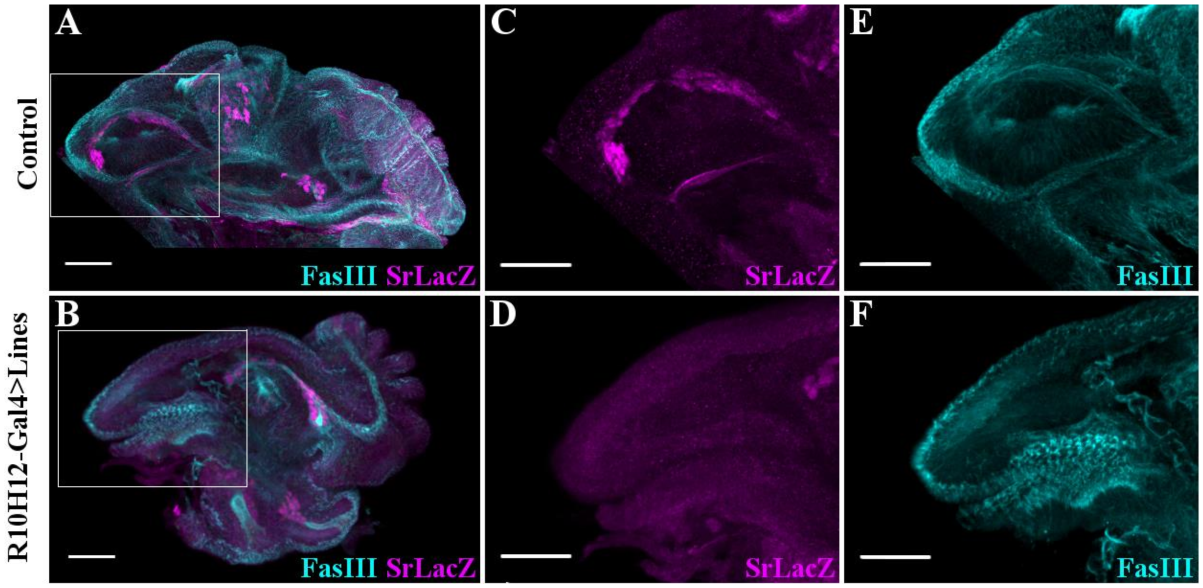


**Figure S4. Expression of *Lines* disrupts *stripe-lacZ* expression**

Confocal sections of R10H12-Gal4 ; Sr-lacZ leg discs at 5h APF immunostained with anti-lacZ (magenta) and anti-FasIII (cyan). (A) Control leg disc and (B) leg disc expressing UAS Lines. (C) and (E) higher magnifications from (A) showing Sr-LacZ expression in cells forming a long internal tube that elongates into the dorsal femur cavity. (D) and (F) Higher magnifications from (B), Sr-LacZ expression is completely abolished in dorsal femur after Lines expression (D); no invaginating tube can be formed from the epithelial cells in the dorsal femur cavity (compare F with E).

Biology Open • Supplementary information

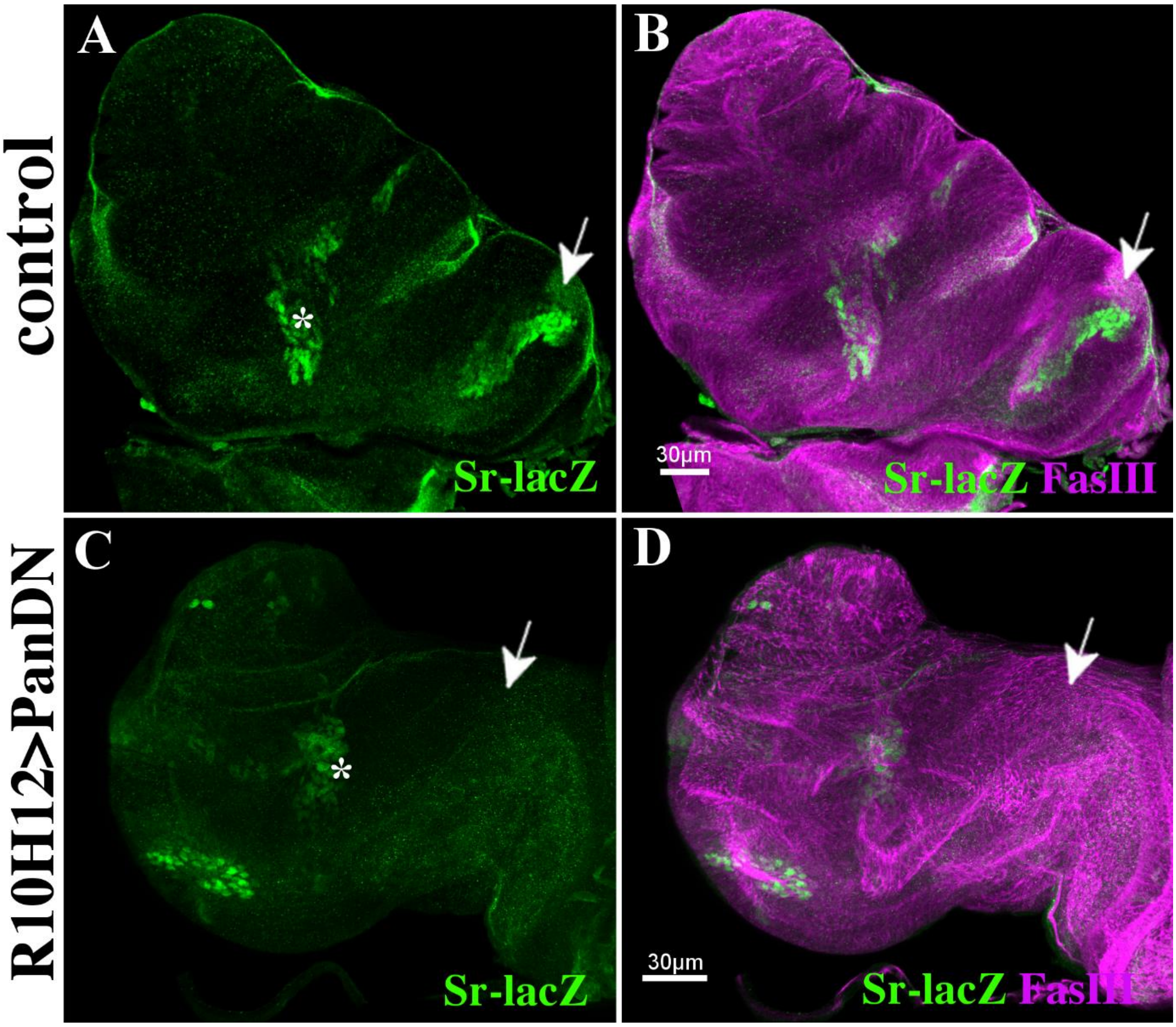


**Figure S5. Inhibition of Wnt pathway disrupts *stripe-lacZ* expression.**

Confocal sections of R10H12-Gal4 ; Sr-lacZ leg discs at 0h APF immunostained with anti-lacZ (green) and anti-FasIII (magenta). (A-B) Control leg disc and (C-D) leg disc expressing UAS-PanDN. In the dorsal femur, Sr-LacZ is completely abolished when a dominant negative form of Pan is expressed using R10H12-gal4 (compare arrows in B and D) whereas Sr-LacZ is still visible in other tendon precursors outside the expression domain of this driver, as it is the case for the long tendon of the tarsi (stars).

Biology Open • Supplementary information
